# Improvement of Late-Onset Hypogonadism Symptoms of Fermented *Morinda citrifolia* Extract in TM3 Leydig and TM4 Sertoli Cells

**DOI:** 10.3390/nu16234159

**Published:** 2024-11-30

**Authors:** Hee-Yeon Kwon, Hyesung Lee, Ji-Soo Choi, Seong Hun Lim, Taehyun Kim, Kyeongseok Bae, Yoon A Jang, Jae-Yeon Lee, Se-Young Choung, Do Sik Min

**Affiliations:** 1 College of Pharmacy, Yonsei University, Songdogwahak-ro, Yeonsu-gu, Incheon 21983, Republic of Korea; khy@nstbio.co.kr (H.-Y.K.); hyesung.lee@yonsei.ac.kr (H.L.); cjs116@nstbio.co.kr (J.-S.C.); limhun19@naver.com (S.H.L.); alpha990505@gmail.com (T.K.); bks1999@naver.com (K.B.); dbsdk0720@gmail.com (Y.A.J.); 2 R&D Center, NSTbio Co., Ltd., 32 Songdogwahak-ro, Yeonsu-gu, Incheon 21984, Republic of Korea; jyleebio@nstbio.co.kr; 3 Department of Preventive Pharmacy, College of Pharmacy, Dankook University, Cheonan 31116, Republic of Korea; sychoung@dankook.ac.kr

**Keywords:** late-onset hypogonadism, *Morinda citrifolia*, fermentation, TM3 Leydig cells, TM4 Sertoli cells, hydrogen peroxide, oxidative stress

## Abstract

Background/Objectives: Late-onset hypogonadism (LOH), characterized by declining testosterone levels with age, negatively affects the health of men, causing physical, psychological, and sexual dysfunction. Conventional testosterone replacement therapies have side effects, which has led to interest in natural alternatives. We investigated the effects of a standardized fermented *Morinda citrifolia* extract (FME) on oxidative stress-induced damage in TM3 Leydig and TM4 Sertoli cells. The cells were treated with H_2_O_2_ to simulate oxidative stress, followed by the FME treatment. Methods: Cytotoxicity assays, testosterone measurements, and gene and protein expression analyses were conducted to evaluate the restorative properties of FME. Results: The H_2_O_2_ treatment significantly decreased the cell viability, testosterone production, and the expression of proteins involved in testosterone synthesis and spermatogenesis, and the FME treatment improved testosterone production and restored the luteinizing hormone receptor, steroidogenic acute regulatory protein, CYP11A1, 3β-hydroxysteroid dehydrogenase, 17,20 desmolase, and 17β-hydroxysteroid dehydrogenase levels in the TM3 Leydig cells. It also reduced the expression of testosterone-degrading enzymes, aromatase and 5α-reductase. The FME treatment restored the levels of the androgen receptor and follicle-stimulating hormone receptor in the TM4 Sertoli cells. Conclusions: FME alleviates oxidative stress-induced damage in Leydig and Sertoli cells by promoting testosterone synthesis and spermatogenesis while regulating testosterone metabolism. These findings suggest that FME could be a promising candidate for the management of LOH symptoms.

## 1. Introduction

Developments in medical science have led to an increase in the average human lifespan, resulting in a growing ratio of elderly individuals in the population and a rapidly evolving awareness regarding their health [1]. As the average age of societies rises, there is not only a fundamental need for healthcare but also an increasing desire to maintain vitality and health during middle and old age, by preventing or delaying the aging process [2]. Menopausal symptoms, resulting from endocrine hormone imbalances owing to aging, are known to significantly impact the quality of life of the elderly [3]. The terminology for male menopause began in 1939 with Werner, who first defined it as ‘male climacteric’ [4]. Since then, various terms such as ‘male menopause’, ‘andropause’, ‘penopause’, and ‘partial androgen deficiency of the aging male (PADAM)’ have been used. More recently, it has been defined as ‘late-onset hypogonadism (LOH)’, which is now commonly employed in academic discourse [5]. LOH has a detrimental impact on male health in middle and older age, accelerating aging and reducing overall physiological resilience [6]. Common symptoms of LOH and menopause include fatigue, poor memory, frequent depression, and physical issues such as decreased muscle strength, increased body fat, and weakened bones [7]. Sexual symptoms include decreased sexual function, erectile dysfunction, low libido, nervousness, and sweating [8]. Female menopause, caused by a rapid decrease in estrogen levels, is more noticeable because of a sudden drop in hormone levels [9]. In contrast, early symptoms of LOH are less apparent because of the gradual decline in testosterone levels, often worsening owing to neglect [9,10].

Testosterone, produced by Leydig cells in the testes, promotes sperm formation through steroidogenesis initiated by the steroidogenic acute regulatory protein (StAR) [11,12]. When luteinizing hormone (LH) from the anterior pituitary binds to the luteinizing hormone receptor (LHR) and activates the signal, StAR mobilizes cholesterol to the inner mitochondrial membrane of Leydig cells [13]. Free cholesterol in the inner mitochondrial membrane is converted to pregnenolone by cytochrome P450 (P450scc or CYP11A1) [14]. Pregnenolone is then converted to progesterone by the 3β-hydroxysteroid dehydrogenase 2 (3β-HSD2) enzyme in the endoplasmic reticulum, which is further converted to androstenedione by P450c17 (17α-hydroxylase or 17, 20 desmolase) [15]. Finally, androstenedione is converted into testosterone by 17β-hydroxysteroid dehydrogenase 3 (17β-HSD3) [16]. Testosterone is metabolized into estradiol, a potent estrogen, by the aromatase enzyme, and into dihydrotestosterone (DHT) by the 5α-reductase enzyme [17,18]. As men age, the number of Leydig cells gradually decreases, consequently leading to a decrease in testosterone production [19], which in turn reduces sperm production and causes a decrease in muscle mass and fat metabolism disorders, leading to an increase in fat mass [20]. Sertoli cells in the testicular seminiferous tubules regulate cell differentiation, proliferation, autophagy, and spermatogenesis in the testis by interacting with testosterone and the androgen receptor (AR) [21,22]. Additionally, Sertoli cells have follicle-stimulating hormone receptors (FSHRs) for follicle-stimulating hormone (FSH) from the anterior pituitary, which are crucial for regulating spermatogenesis [23]. Sertoli cell function declines earlier than Leydig cell function, significantly affecting testicular cell differentiation, proliferation, and spermatogenesis [24,25,26].

Research on directly supplementing male hormones via oral administration or injection as a treatment for male menopause has shown controversial outcomes for long-term effectiveness and safety [27,28]. The reported side effects of testosterone supplementation include an increased red blood cell count, sleep apnea, an enlarged prostate, and prostate cancer [29,30,31,32]. Consequently, developing materials from natural substances that promote testosterone production and regulate metabolism is being proposed as an alternative therapy [33,34,35].

Noni (*Morinda citrifolia* L.), a medicinal plant that has been used in Polynesia, India, and China for approximately 2000 years, belongs to the *Rubiaceae* family, which comprises approximately 80 species, with noni considered the “Queen” [36,37]. Noni pulp contains approximately 160 chemical compounds, including phenolic compounds, organic acids, and alkaloids [38]. The components identified in noni include polysaccharides, iridoids, coumarins, anthraquinones, glycosides, triterpenoids, flavonoids, carotenoids, lignans, phytosterols, monoterpenes, short-chain fatty acids, and fatty acid esters [39,40,41]. Ripe noni pulp contains vitamins (vitamins C, A, B1, B2, B3, B6, B12, folic acid, vitamin E, and pantothenic acid), minerals (calcium, phosphorus, magnesium, iron, potassium, and sodium), and dietary fiber [42]. Noni pulp consumption has been reported to have antibiotic, antiviral, antifungal, antitumor, anthelmintic, pain-relieving, hypotensive, anti-inflammatory, low-density lipoprotein (LDL) oxidation-preventing, and immune-boosting effects [43,44]. Recent studies have explored the effects of noni on male reproductive health. One study reported significant increases in testosterone and FSH levels when noni juice was administered to 16-week-old rats for 60 days [45]. Another study showed that oral supplementation with noni extract improved sperm morphology and motility [46]. The oral administration of noni significantly improved testicular tissue damage in a rat model of testicular damage induced by the subcutaneous injection of 3-methyl-4-nitrophenol (PNMC) [47].

To develop health functional foods, standardization must be achieved through the efficacy evaluation and analysis of the active ingredients [48]. Standardization ensures consistent efficacy by minimizing variations in the functional ingredients of natural products [49]. It involves comprehensive quality control from raw materials to final products and maintains changes in indicator components at a consistent level [50]. The fermented *Morinda citrifolia* extract (FME) used in this study has undergone complete material standardization, with a deacetylasperulosidic acid (DAA) component identified as the main phytochemical of noni.

This study aimed to develop a natural source-based functional food material using standardized FME to improve LOH. To investigate the efficacy of FME in improving LOH, its effectiveness and mechanisms of action were evaluated in vitro using mouse-derived Leydig and Sertoli cells. Protein expression involved in testosterone synthesis (LHR, StAR, CYP11A1, 3β-HSD2, 17, 20 desmolase, and 17β-HSD3) and testosterone degradation (5α-reductase and aromatase) was measured in Leydig cells, and the expression of AR and FSHR was measured in Sertoli cells.

## 2. Materials and Methods

### 2.1. Preparation of Fermented Morinda citrifolia Extract (FME) and Testofen

FME was provided by NSTbio Co., Ltd. (Gimpo, Republic of Korea). The *Morinda citrifolia* fruit was sourced from Indonesia. The harvested noni fruit was washed thoroughly and stored at −27 °C for preservation. The frozen noni was thawed at 37 °C for 48 h, inoculated with NST 1805 *Lactiplantibacillus plantarum* culture (2.0% *v*/*w*), and fermented at 37 °C for 60 days. The fermented noni was then squeezed and filtered using filter paper and a vacuum filter (80 mesh) to remove debris and fruit particles. The extract was concentrated to 60 ± 5 Brix, sterilized, and stored at −20 °C for experimental use. The indicator components in the FME were analyzed through an HPLC-UV analysis in the methods of our previous study, and the DAA content of the FME was strictly controlled at 13.0 ± 2.6 mg/g [51]. Testofen, a health functional food material for improving LOH approved by the Korean Ministry of Food and Drug Safety, was used as a positive control to verify the functionality of the FME. Testofen, derived from *Trigonella foenum-graecum* seeds, has been validated through in vitro, in vivo, and clinical trials [52,53].

### 2.2. Cell Culture

TM3 cells (mouse-derived Leydig cells) and TM4 cells (mouse-derived Sertoli cells) were obtained from the Korea Cell Line Bank (Seoul, Republic of Korea) and cultured in Dulbecco’s modified Eagle’s medium (DMEM; HyClone, Logan, UT, USA) supplemented with 10% fetal bovine serum (Gibco BRL, Life Technologies Co., New York, NY, USA) and 1% penicillin-streptomycin (Welgene, Daegu, Republic of Korea). The cells were maintained at 37 °C with 5% CO_2_ and subcultured every 3 days. Oxidative stress was induced by replacing the medium with a serum-free medium supplemented with 100 μM hydrogen peroxide (Sigma-Aldrich, St. Louis, MO, USA) and culturing for 4 h. Subsequently, the cells were treated with a medium containing the respective concentrations of FME or Testofen, per experimental design, and cultured for 24 h, after which the cells and medium were collected for further experiments.

### 2.3. Cell Viability Assay

To evaluate FME cytotoxicity, TM3 Leydig and TM4 Sertoli cells were seeded in 96-well plates at a density of 5 × 10^4^ cells/0.2 mL/well and cultured for 48 h. The medium was then replaced with a medium containing various concentrations of FME (50, 100, 200, 500, and 1000 μg/mL) and Testofen (12.5, 25, 50, 100, and 200 μg/mL) (BTC, Ansan, Republic of Korea), and the cells were cultured for an additional 24 h. To assess the cytotoxicity of hydrogen peroxide, cells were similarly seeded and cultured, replaced with serum-free DMEM containing different concentrations of hydrogen peroxide (6.25, 12.5, 25, 50, 100, 200, 500, and 1000 μM) and cultured for 4 h. The measurements were performed using the CellTiter 96^®^ AQueous One Solution Cell Proliferation Assay kit (Promega, Madison, WI, USA). The absorbance was measured at 490 nm using an ELISA reader (BioTek Instruments Inc., Winooski, VT, USA). The cytotoxicity was calculated relative to untreated control cells.

### 2.4. Measurement of Testosterone Level

To investigate the impact of H_2_O_2_ on testosterone production, TM3 Leydig cells were seeded in a six-well plate at a density of 5 × 10^5^ cells/2 mL/well and cultured for 48 h. Subsequently, the cells were exposed to varying concentrations of H_2_O_2_ (50, 100, 200, 500, and 1000 μM) for 4 h in serum-free DMEM. The medium was then replaced with DMEM containing 10% FBS, and the cells were cultured for an additional 24 h. Supernatants were collected for testosterone analysis. To assess the effect of FME on testosterone production in Leydig cells that had been subjected to oxidative stress by exposure to H_2_O_2_, TM3 Leydig cells were seeded in a six-well plate at a density of 5×10^5^ cells/2 mL/well and cultured for 48 h. The medium was then replaced with a serum-free medium supplemented with 100 μM H_2_O_2_ and cultured for 4 h to induce oxidative stress. Following this, the medium was replaced with a medium containing various concentrations of FME (50, 200, and 500 μg/mL), the cells were cultured for an additional 24 h, and supernatants were collected for testosterone analysis. Testosterone levels in the supernatant were measured using a testosterone ELISA kit (ElabSciences, Houston, TX, USA), according to the manufacturer’s protocol.

### 2.5. Quantitative Real-Time PCR(qRT-PCR) Analysis

The qRT-PCR experiment was conducted according to previously published methods [54]. Ttotal RNA was isolated from the cells using an easy-Blue kit (iNtRON, Seongnam, Republic of Korea), and cDNA was synthesized using a High-Capacity RNA-to-cDNA kit (Thermo Fisher Scientific, Waltham, MA, USA). Template DNA was extracted using the AccuPrep Genomic DNA Extraction Kit (Bioneer, Daejeon, Republic of Korea). Quantification was conducted over 40 cycles with the following incubation conditions: 15 min at 48 °C, 10 min at 95 °C, 15 s at 95 °C, and 1 min at 60 °C. Data analysis was performed using the StepOne Software v2.3. The expression of the housekeeping gene *GAPDH* was normalized using a mathematical model of relative expression ratios, which accounted for PCR efficiency. The primer sequences are presented in Table 1.

### 2.6. Western Blot Analysis

Cells were homogenized in a lysis buffer containing the cOmplete™ protease inhibitor cocktail (Roche Diagnostics, Indianapolis, IN, USA) and 5 μM EDTA (Sigma-Aldrich, Oakville, ON, Canada). The supernatant was centrifuged at 12,000 rpm at 4 °C. Protein concentration was determined using a BCA protein assay kit (Bio-Rad, Hercules, CA, USA). Lysates were subjected to sodium dodecyl sulfate-polyacrylamide gel electrophoresis. After the electrophoresis, the proteins were transferred to a PVDF membrane, and the membrane was blocked with 5% skim milk and incubated overnight with primary antibodies. The primary antibodies used were LHR (Bioss, Beijing, China), StAR, CYP11A1, GAPDH (Cell Signaling, Danvers, MA, USA), 3β-HSD2, FSHR (Mybiosource, San Diego, CA, USA), 17,20 desmolase (Proteintech, Rosemont, IL, USA), 17β-HSD3, aromatase, 5α-reductase (Invitrogen, Eugene, OR, USA), and AR (Santa Cruz Biotechnology, Dallas, CA, USA). The primary antibodies were reacted with the corresponding secondary antibodies and protein bands were visualized using the ChemiDoc MP Imaging System (Bio-Rad, Hercules, CA, USA). Secondary anti-rabbit and anti-mouse antibodies were purchased from Santa Cruz Biotechnology. GAPDH was used as a loading control and quantitative analysis was performed using ImageJ software (version 1.52a, National Institutes of Health, Bethesda, MD, USA).

### 2.7. Measurement of 3β-HSD2, 17β-HSD3, 5α-Reductase, and Aromatase Levels

To investigate the effect of FME on the expression of enzymes related to testosterone production and degradation, TM3 Leydig cells were seeded in six-well plates at a density of 5 × 10^5^ cells/2 mL/well and cultured for 48 h. The medium was then replaced with serum-free medium supplemented with 100 μM hydrogen peroxide to induce oxidative stress for 4 h. Subsequently, the medium was replaced with DMEM supplemented with various concentrations of FME, and the cells were cultured for an additional 24 h. The cells were collected, and the enzyme expression was measured using ELISA kits for 3β-HSD2, 17β-HSD3, 5α-reductase, and aromatase (MyBioSource, San Diego, CA, USA) according to the manufacturer’s protocol.

### 2.8. Statistical Analysis

Results are expressed as the mean ± standard deviation (SD). Statistical significance was determined with Student’s *t*-test using Microsoft Excel 2016. Statistical significance was set at *p* < 0.05, marked with a single asterisk (*). *p*-values below 0.01 and 0.001 are denoted with a double asterisk (**) and triple asterisk (***), respectively.

## 3. Results

### 3.1. Effect of FME, Testofen, and H_2_O_2_ on the Viability of TM3 and TM4 Cells

**Figure 1:**
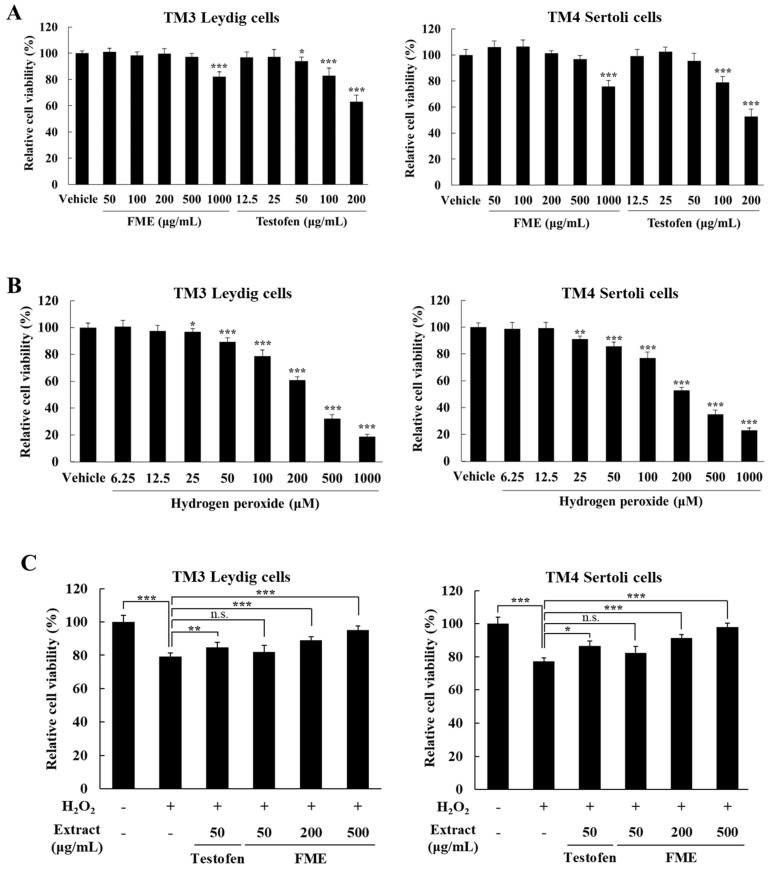
Effect of fermented *Morinda citrifolia* extract (FME), Testofen, and H_2_O_2_ on the viability of TM3 Leydig and TM4 Sertoli cells. TM3 and TM4 cells were treated with the indicated concentration of FME (50–1000 μg/mL) or Testofen (12.5–200 μg/mL) for 24 h with DMEM containing 10% FBS (**A**), and the indicated concentration of H_2_O_2_ for 4 h with serum-free DMEM (**B**). TM3 cells were treated with H_2_O_2_ (100 μM) for 4 h in serum-free DMEM, and then with FME for 24 h (**C**). Cell viability was measured using an MTS assay. The results were expressed as the mean ± SD (n = 6). * *p* < 0.05, ** *p* < 0.01, *** *p* < 0.001 significantly different from vehicle group. n.s.: not significant.

A cytotoxicity assay was conducted to determine the appropriate extract concentrations to be used in the experiments. The cells were treated with FME at 50, 100, 200, 500, and 1000 μg/mL, and Testofen, serving as a positive control, at 12.5, 25, 50, 100, and 200 μg/mL. Cell viability was assessed after 24 h of culture (Figure 1A). No significant differences in cell viability were observed between the vehicle group and the FME-treated groups (50–500 μg/mL) in both TM3 Leydig and TM4 Sertoli cells, indicating that FME has no cytotoxic effect on these cells below 500 μg/mL (*p* > 0.05) (Figure 1A). Consequently, concentrations up to 500 μg/mL were used to evaluate the efficacy in LOH. The Testofen-treatment groups exhibited significant cytotoxicity at concentrations of 100 and 200 μg/mL in both TM3 and TM4 cells compared with the vehicle group (*p* < 0.001) (Figure 1A). Although slight cytotoxicity was observed at 50 μg/mL of Testofen in TM3 cells, the viability remained at 93.91 ± 4.57%, which was not deemed to have a substantial impact on experimental results. For establishing an oxidative stress-induced in vitro model pertinent to LOH research, cells were treated with H_2_O_2_ at concentrations of 6.25, 12.5, 25, 50, 100, 200, 500, and 1000 μM and cultured for 4 h. The cell viability significantly decreased in both TM3 and TM4 cells upon the H_2_O_2_ treatment, with notable effects starting from 25 μM (*p* < 0.05). To induce oxidative stress, a concentration of 100 μM H_2_O_2_, which reduces the cell viability by 21.34%, was selected for the subsequent evaluations of the LOH efficacy (Figure 1B).

To assess the cell viability recovery potential of FME in TM3 and TM4 cells that had been exposed to H_2_O_2_-induced oxidative stress, the cells were treated with 100 μM H_2_O_2_ for 4 h, followed by FME treatment (50, 200, and 500 μg/mL) for 24 h. The FME treatment at concentrations of 200 and 500 μg/mL significantly recovered the viability of both TM3 and TM4 cells reduced by the H_2_O_2_ treatment (Figure 1C). Compared to the group treated with 100 μM H_2_O_2_ alone, the group treated with 500 μg/mL FME significantly restored cell viability by 20.35% in TM3 cells and 26.74% in TM4 cells (*p* < 0.001).

### 3.2. FME Enhances Testosterone Production in H_2_O_2_-Treated TM3 Cells

**Figure 2:**
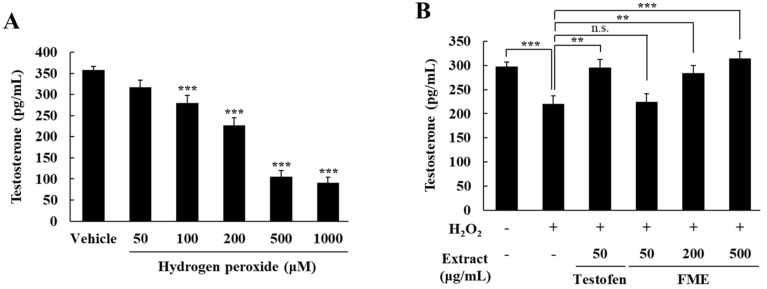
Effect of H_2_O_2_ and FME on testosterone production in TM3 Leydig cells. (**A**) H_2_O_2_, (**B**) FME and Testofen treatment following H_2_O_2_ exposure. TM3 cells were treated with H_2_O_2_ (100 μM) for 4 h in serum-free DMEM. Then, the cells were treated with the indicated concentration of FME for 24 h. After this, testosterone levels were measured in the supernatant using a testosterone ELISA kit. The results are expressed as the mean ± SD (n = 6). ** *p* < 0.01, *** *p* < 0.001 significantly different from vehicle group. n.s.: not significant.

To assess the impact of H_2_O_2_ on testosterone production, TM3 Leydig cells were treated with varying concentrations of H_2_O_2_ (50, 100, 200, 500, and 1000 μM) for 4 h in serum-free DMEM. The testosterone levels secreted from Leydig cells significantly decreased with increasing H_2_O_2_ concentrations, with a statistically significant difference being observed starting at a concentration of 100 μM (*p* < 0.001) (Figure 2A). To evaluate the effect of FME on testosterone production, oxidative stress was induced in TM3 cells by treating them with 100 μM H_2_O_2_ for 4 h. Subsequently, the cells were treated with various concentrations of FME (50, 200, and 500 μg/mL), and the testosterone levels were measured after 24 h. Compared with the vehicle control group, the testosterone production decreased by 26.12% in the H_2_O_2_-treated groups (*p* < 0.001), whereas the subsequent FME treatment resulted in increases of 2.00%, 29.25%, and 42.98% at 50, 200, and 500 μg/mL, respectively, compared with the H_2_O_2_-treated group (Figure 2B). The Testofen treatment group, used as a positive control, also restored the reduced testosterone production caused by H_2_O_2_. Notably, the FME (500 μg/mL) treatment group demonstrated a 106.6% increase in testosterone secretion compared to the Testofen group. These findings indicate that the FME treatment enhanced the testosterone production in TM3 cells in a concentration-dependent manner.

### 3.3. FME Modulates the mRNA Expression of Genes Involved in the Synthesis and Degradation of Testosterone

**Figure 3:**
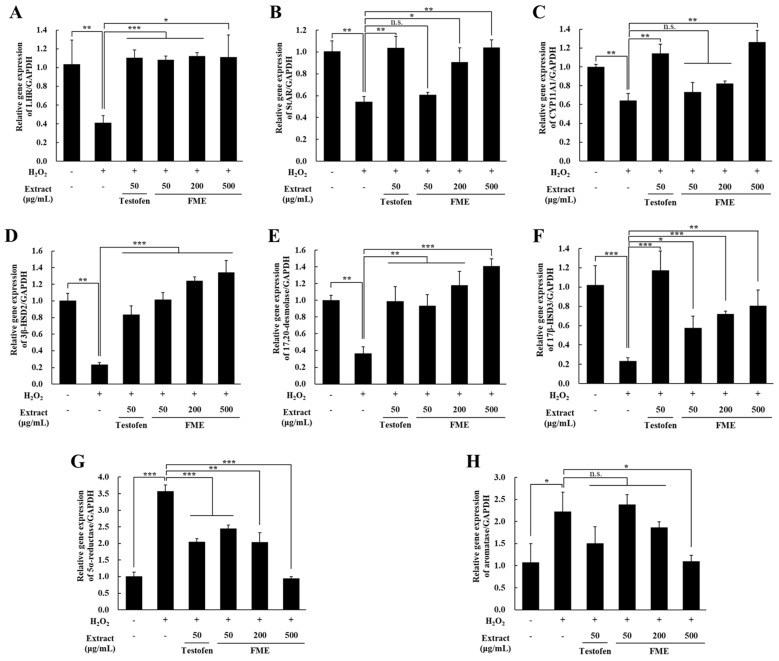
Effect of FME on mRNA expression in H_2_O_2_-treated TM3 Leydig cells. TM3 cells were treated with H_2_O_2_ (100 μM) for 4 h in serum-free DMEM. Then, the cells were treated with the indicated concentration of FME for 24 h. After this, RNA was extracted from TM3 cells. mRNA expression of the (**A**) luteinizing hormone receptor (*LHR*), (**B**) steroidogenic acute regulatory protein (*StAR*), (**C**) *CYP11A1*, (**D**) *3β-HSD2*, (**E**) *17,20 desmolase*, (**F**) *17β-HSD3*, (**G**) 5α-reductase, and (**H**) aromatase was measured using qRT-PCR. The results are expressed as the mean ± SD (n = 3). * *p* < 0.05, ** *p* < 0.01, *** *p* < 0.001 significantly different from H_2_O_2_-only treated group. n.s.: not significant.

To examine whether FME affects the expression of genes related to testosterone synthesis and degradation in Leydig cells, TM3 cells under oxidative stress were treated with FME for 24 h, followed by a qRT-PCR analysis. *LHR* expression, which was reduced by H_2_O_2_, was significantly recovered by both Testofen and FME treatments (Figure 3A). *StAR* expression was decreased by H_2_O_2_ and was also reversed by the FME (Figure 3B). In addition, the mRNA expression of *CYP11A1*, *3β-HSD2*, *17,20 desmolase*, and *17β-HSD3*, which are involved in testosterone biosynthesis, was decreased by the treatment with H_2_O_2_, but significantly recovered with the FME treatment (*p* < 0.05) (Figure 3C–F). Conversely, the expression of 5α-reductase and aromatase, that metabolize testosterone, was increased with the H_2_O_2_ treatment, but decreased with the FME treatment (*p* < 0.05) (Figure 3G,H). Notably, the mRNA expression of 5α-reductase and aromatase in the 500 μg/mL FME-treated group were reduced to levels comparable with those of the untreated group (*p* > 0.05). Compared to the Testofen treatment group, the FME (500 μg/mL) treatment group increased the expression of LHR, StAR, CYP11A1, 3β-HSD2, and 17,20-desmolase, while reducing the gene expression of 5α-reductase and aromatase.

### 3.4. Effect of FME on Protein Expression of Testosterone Synthesis or Degradation Biomarkers

**Figure 4:**
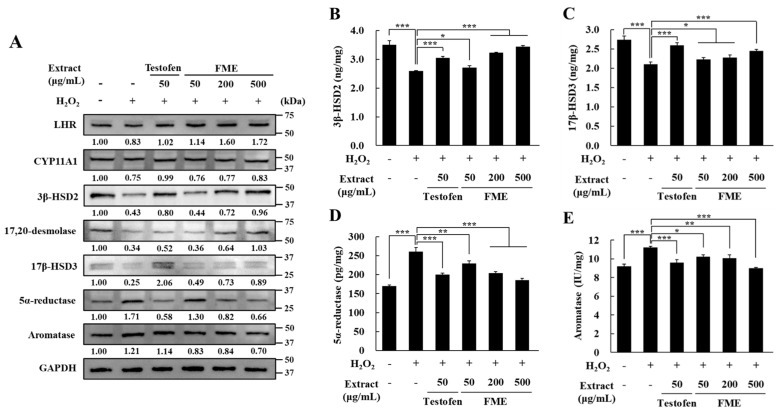
Effect of FME on protein expression in H_2_O_2_-treated TM3 Leydig cells. TM3 cells were treated with H_2_O_2_ (100 μM) for 4 h in serum-free DMEM. Then, the cells were treated with the indicated concentration of FME for 24 h. After this, the proteins were extracted from the TM3 cells. The protein expression of LHR, StAR, CYP11A1, 3β-HSD2, 17,20 desmolase, 17β-HSD3, 5α-reductase, and aromatase was measured using (**A**) western blot, and (**B**) 3β-HSD2, (**C**) 17β-HSD3, (**D**) 5α-reductase, and (**E**) aromatase were measured using ELISA kits. Values are expressed as the mean ± SD (n = 6). * *p* < 0.05, ** *p* < 0.01, *** *p* < 0.001 significantly different from the H_2_O_2_-only treated group.

To further verify the effects of FME on the levels of proteins involved in testosterone synthesis and degradation in Leydig cells, TM3 cells that had been subject to oxidative stress were treated with FME for 24 h. The cells were then collected, and protein levels were assessed using western blotting and ELISA. The LHR expression decreased by 17% after the H_2_O_2_ treatment but recovered to levels comparable to those in the untreated group after the Testofen and FME treatments (Figure 4A). The protein expressions of CYP11A1, 3β-HSD2, 17,20 desmolase, and 17β-HSD3, enzymes involved in testosterone biosynthesis, decreased with the H_2_O_2_ treatment but increased in a concentration-dependent manner with the FME treatment (Figure 4A). The protein expression of 5α-reductase and aromatase increased with the H_2_O_2_ treatment and was reduced in a concentration-dependent manner with the FME treatment (Figure 4A). Furthermore, ELISA showed that the protein level of 3β-HSD2 decreased by 26.17% with the H_2_O_2_ treatment, which was significantly recovered with the FME treatment (*p* < 0.05) (Figure 4B). Additionally, the protein expression of 17β-HSD3 decreased by 23.28% with the H_2_O_2_ treatment and was also significantly recovered with the FME treatment (*p* < 0.05) (Figure 4C). Moreover, the protein levels of 5α-reductase and aromatase were increased by 53% and 22% with the H_2_O_2_ treatment, respectively, but significantly decreased with the FME treatment (*p* < 0.01) (Figure 4D,E). Comparison of protein expression levels between the Testofen and FME treatment groups revealed that the FME (500 μg/mL) group induced a higher increase in LHR, 3β-HSD2, and 17,20-desmolase expression, while decreasing the expression of aromatase.

### 3.5. FME Regulates the Expression Levels of AR and FSHR in Sertoli Cells

**Figure 5:**
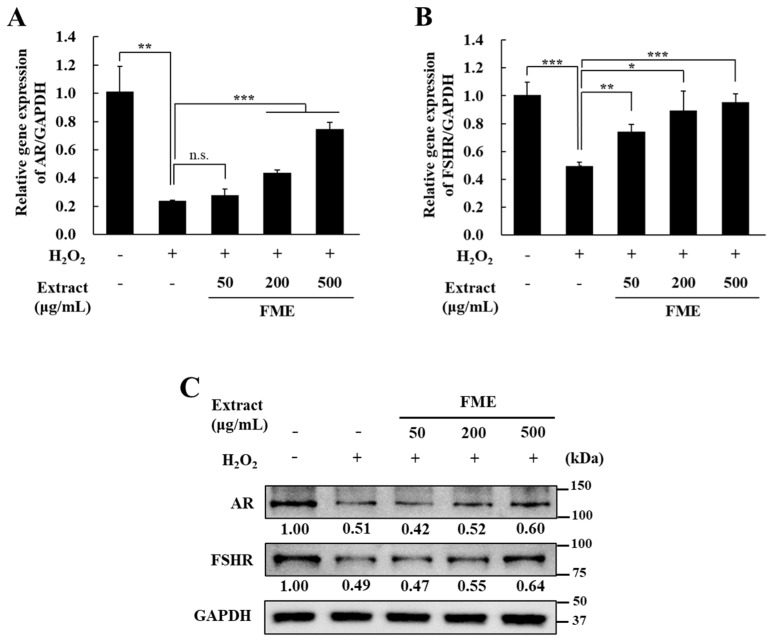
Effect of FME on the expression level of the androgen receptor (AR) and follicle stimulating hormone receptor (FSHR), evaluated using (**A**,**B**) qRT-PCR and (**C**) western blotting in H_2_O_2_-treated TM4 Sertoli cells. TM4 cells were treated with H_2_O_2_ (100 μM) for 4 h in serum-free DMEM. Then, the cells were treated with FME (50, 200, and 500 μg/mL) for 24 h. After this, RNA and protein were extracted from TM4 cells. Values are expressed as the mean ± SD (n = 3). * *p* < 0.05, ** *p* < 0.01, *** *p* < 0.001 significantly different from H_2_O_2_-only treated group. n.s.: not significant.

To measure the AR and FSHR levels, the key receptors related to male reproduction in Sertoli cells, both mRNA and protein expression levels were evaluated using qRT-PCR and western blotting, respectively. When oxidative stress was induced in TM4 cells using H_2_O_2_, the mRNA level of the AR decreased by 76.30% compared with untreated cells, but significantly increased with the FME treatment (Figure 5A). The FSHR mRNA expression decreased by 50.60% after the H_2_O_2_ treatment compared with the untreated cells, and significantly increased after the FME treatment (*p* < 0.05) (Figure 5B). The AR protein levels showed a 49.0% decrease following the H_2_O_2_ treatment compared with the untreated cells, which increased in a concentration-dependent manner following the FME treatment. Similarly, the FSHR protein levels were reduced by 51.0% with the H_2_O_2_ treatment compared with the untreated cells and increased in a concentration-dependent manner with the FME treatment.

## 4. Discussion

The direct supplementation of testosterone is commonly employed to treat sexual dysfunction and LOH symptoms [27,28]. However, recent reports have highlighted various side effects associated with these treatments, prompting a shift towards health functional foods with a lower risk profile [55]. *Morinda citrifolia*, known for its rich phytochemical content, has gained attention in recent studies focused on male health [45,46,47]. Notably, the FME used in this study is a standardized preparation with an enhanced content of DAA, the primary active compound in noni [48].

The main cause of LOH symptoms is a decline in testosterone levels, which is closely linked to Leydig cell functionality. A decrease in Leydig cells can lead to male infertility by reducing testosterone levels and by significantly affecting sperm quality and motility [11,19]. Reactive oxygen species (ROS) are also generated during the metabolic processes of Leydig cells and occur in various membrane-bound organelles, such as the endoplasmic reticulum and mitochondria [56]. Enzymes involved in the steroidogenesis cycle, which facilitate the loss of electrons (oxidation) from metabolic byproducts, act as sources of free radical generation, and it has also been reported that stimulation by LH increases ROS production [57]. As men age, their defense mechanisms against oxidative stress weaken, making cells more susceptible to damage from oxidative stress induced by aging, inflammation, and environmental hormones [19,58,59]. Many studies have identified oxidative stress-induced apoptosis as one of the primary causes of the reduced quantity and functionality of Leydig cells [60,61,62,63]. Consequently, oxidative stress-induced Leydig cells are commonly used as an in vitro model for LOH research [52,60,61,63,64,65,66].

In this study, we subjected mouse-derived TM3 Leydig and TM4 Sertoli cells to oxidative stress via H_2_O_2_ treatment. We treated cells with H_2_O_2_ using serum-free media, which does not contain FBS, to induce oxidative stress. FBS contains various components essential for cell culture, some of which possess antioxidant activity [67]. For example, bilirubin and albumin have been reported to exhibit antioxidant effects in both in vitro and in vivo studies [68,69,70,71,72]. Thus, to accurately assess the effects of pure H_2_O_2_ in oxidative stress studies, it is important to use serum-free media to eliminate any potential interference from the antioxidant components in FBS. Initially, we evaluated the cytotoxic effects of FME and Testofen on TM3 and TM4 cells. The FME-treated group exhibited no toxicity up to 500 μg/mL, whereas the Testofen-treated group showed significant cytotoxicity starting at 100 μg/mL. These findings align with those of Kim et al. (2019), who reported Testofen toxicity in TM3 and TM4 cells at 100 μg/mL, whereas the results of this study showed that FME is safer at higher concentrations [52]. A concentration of 100 μM H_2_O_2_, commonly used to induce oxidative stress in LOH studies, typically reduces cell viability by approximately 20–25% [35,73,74]. Similarly, in our study, treatment with 100 μM H_2_O_2_ reduced cell viability by approximately 20%, which was significantly restored by the FME treatment. Previous reports have indicated that *Morinda citrifolia* fruit extracts can neutralize oxidative stress in a dose-dependent manner, owing to their polyphenol content [75]. Additionally, *Morinda citrifolia* juice has been shown to improve oxidative stress by upregulating antioxidant enzymes, such as superoxide dismutase and glutathione peroxidase, and reducing the levels of reactive oxygen species [76]. Our results demonstrated that FME protects and restores Leydig and Sertoli cells from oxidative damage.

Testosterone is secreted by testicular Leydig cells, and damage to these cells can lead to decreased testosterone levels [77]. FME treatment significantly restored the testosterone levels diminished by H_2_O_2_ in TM3 Leydig cells and enhanced testosterone secretion beyond the levels observed in the Testofen-treated group at non-toxic concentrations. In this study, the high-dose FME treatment group (500 μg/mL) showed a 42.98% increase in testosterone production compared to the H_2_O_2_-only treatment group. A study investigating the LOH improvement effects of *Dendropanax morbiferus* leaf extract (DME) reported a 41.4% increase in testosterone production in TM3 Leydig cells when treated with the highest dose (100 μg/mL) of DME [61]. Similarly, another study using *Eurycoma longifolia* extract reported a 16% increase in testosterone production compared to the H_2_O_2_ treatment group [78]. Notably, FME demonstrated 106.6% of the testosterone production level compared to Testofen, a Ministry of Food and Drug Safety-approved extract for LOH improvement, in this study. This suggests that FME may be a more effective material at enhancing testosterone levels at the safe maximum concentration in cellular models compared to previously reported extracts.

To elucidate the mechanism behind the enhancement of Leydig cell activity by FME, we measured the expression of key proteins: LHR, StAR, CYP11A1, 3β-HSD2, 17,20 desmolase, 17β-HSD3, 5α-reductase, and aromatase. LHR activation by LH from the anterior pituitary increases cAMP levels and activates protein kinase A, boosting StAR and steroidogenic enzyme expression, thereby promoting testosterone synthesis [79]. The increase in LHR observed in this study may enable Leydig cells to respond more sensitively to external stimuli, which may increase their functional activity. The expression of LHR is significantly reduced when TM3 Leydig cells are treated with sodium fluoride [80]. Mice exposed to sodium fluoride are susceptible to oxidative stress due to the induction of an oxidative biochemical imbalance [81]. In addition, rats exposed to perfluorodecanoic acid (PFDoA) were reported to have a significant decrease in the expression of LHR in Leydig cells, and PFDoA was reported to cause toxicity by inducing oxidative stress at the cellular and molecular levels [82,83]. These studies suggest that oxidative stress may reduce LHR levels in Leydig cells. In this study, we found that the expression levels of LHR genes and proteins were significantly reduced by the H_2_O_2_ treatment, and the FME treatment restored them to the same level as in the H_2_O_2-_untreated group. StAR, a protein that transports cholesterol to the inner mitochondrial membrane, is crucial for steroidogenesis [79]. A previous study demonstrated a 50% reduction in StAR protein expression in mouse Leydig cells upon treatment with H_2_O_2_ [67]. Our study corroborated these findings with a similar reduction in StAR expression following H_2_O_2_ treatment, which was significantly reversed by FME, indicating its role in promoting mitochondrial cholesterol metabolism. CYP11A1 is located inside the mitochondria and converts free cholesterol to pregnenolone in the inner mitochondrial membrane through StAR [84]. 3β-HSD2, 17,20 desmolase, and 17β-HSD3 are key enzymes involved in synthesizing testosterone from pregnenolone produced in mitochondria and are important indicators commonly measured in LOH studies [52,65,66,85]. The expression level of enzymes related to testosterone biosynthesis, such as CYP11A1, 3β-HSD2, 17,20 desmolase, and 17β-HSD3, was significantly decreased by H_2_O_2_ treatment, subsequently lowering testosterone production. FME treatment significantly increased the expression of these enzymes, thereby enhancing testosterone production. Byeon et al. (2019) reported that when TM3 cells damaged by H_2_O_2_ were treated with *Lespedeza cuneata* extract, the ability to produce testosterone was restored, and the mechanism of action involved the restoration of enzymes such as CYP11A1, 3β-HSD2, 17,20 desmolase, and 17β-HSD3 [35].

5α-Reductase is an enzyme that metabolizes testosterone to DHT, and both testosterone and DHT interact with AR to regulate androgen-dependent transcription [86]. DHT is a stronger androgen because of its higher affinity for the AR [87,88]. Therefore, testosterone and DHT are important hormones for male reproduction, including spermatogenesis [89]. However, during LOH, the balance of DHT in the body is disrupted, causing various side effects; excess DHT causes hair loss and prostatic hyperplasia [90,91]. Aromatase metabolizes testosterone into estradiol (E2), a potent estrogen [17]. Under normal conditions, E2 is a hormone that plays an important role in male reproductive function, bone growth, and skeletal maintenance [92]. However, with aging, testosterone levels decrease, leading to hormonal imbalances [93]. This hormonal imbalance reduces sperm motility, LH and FSH levels, and fertility, and increases the risk of arterial diseases [94,95]. Therefore, studies are being conducted with the goal of reducing the activity of 5α-reductase and aromatase in LOH. 5α-reductase and aromatase are increased under oxidative stress conditions, which is consistent with the significant increase observed after the H_2_O_2_ treatment in this study [52,65,66,85]. FME treatment significantly reduced the concentration of testosterone-metabolizing enzymes, suggesting that it could improve male menopause by controlling the production of DHT and E2 in Leydig cells.

Spermatogenesis is an elaborately organized process involving many molecules, such as the follicle-stimulating hormone (FSH) and androgens. In adult men, FSH is involved in the proliferation of spermatogonia and production of normal sperm cells [96]. FSH acts through a specific receptor (FSHR) located on the surface of Sertoli cells, which play a major role in male reproduction [97]. FSHR-knockout mice have a reduced testicular size, sperm concentration, and motility, and consequently reduced fertility [98]. Androgens play an important role in the development of male secondary sexual characteristics and in the stimulation and maintenance of spermatogenesis [99]. Androgens regulate a wide range of reproductive processes, including sperm maturation, by binding to the AR [100]. AR-knockout male mice have been reported to have a reduced testicular size and sperm motility [101,102]. Thus, the AR and FSHR in the Sertoli cells perform major functions in male reproduction. We found that the expression of the AR and FSHR was significantly reduced by H_2_O_2_-induced oxidative stress in TM4 Sertoli cells. Zhao et al. (2023) reported that when di-2-ethylhexyl phthalate (DEHP), one of the phthalates, was administered to rats, the expression levels of AR and FSHR were significantly decreased [103]. DEHP induces cell damage via oxidative stress [104]. AR and FSHR can be reduced by oxidative stress, and when FME was used to treat damaged TM4 cells, the levels of the two proteins increased significantly. Therefore, our study suggests that FME can potentially improve male reproductive health during aging by increasing the AR and FSHR levels in Sertoli cells.

Fermentation enhances the concentration of bioactive compounds, including polyphenols, which possess notable antioxidative properties. As noted in previous research, fermentation significantly increases the bioavailability and functional potential of phenolic compounds by releasing bound forms and converting them into bioactive metabolites [105]. This transformation contributed to the observed improvement in LOH symptoms in this study, supporting the hypothesis that FME exerts its effects through enhanced antioxidative activity and the modulation of key bioactive components. Identifying the specific compounds in *Morinda citrifolia* that are transformed during fermentation and determining which of these contribute to the improvement of LOH symptoms is deemed highly important for future research.

## 5. Conclusions

**Figure 6:**
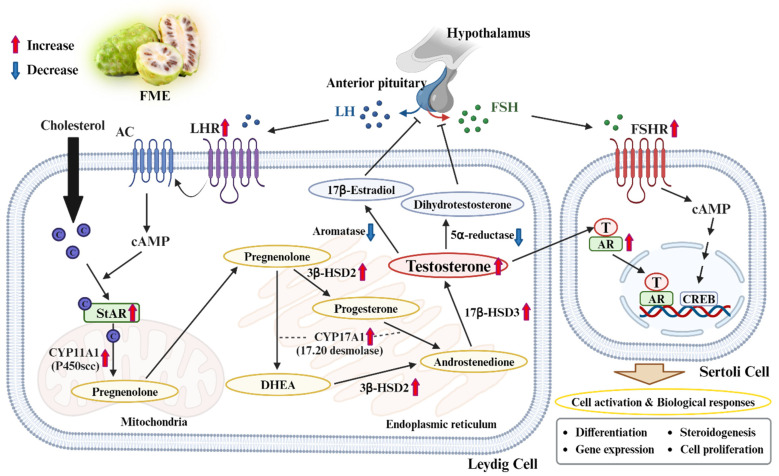
Proposed mechanism of FME in the testosterone metabolism pathway. AC: adenylyl cyclase; cAMP: cyclic adenosine monophosphate; DHEA: dehydroepiandrosterone; CREB: cyclic AMP-responsive element binding protein. Created with Biorender.com.

This study reports the mechanism underlying the effects of FME on LOH by improving testosterone biosynthesis, suppressing its degradation in Leydig cells, and regulating male reproduction-related proteins in Sertoli cells (Figure 6). Our study demonstrated that FME effectively mitigates oxidative stress–induced damage in Leydig and Sertoli cells. This protection is evidenced by the restoration of key proteins involved in testosterone biosynthesis and spermatogenesis, such as LHR, StAR, CYP11A1, 3β-HSD2, 17,20 desmolase, 17β-HSD3, AR, and FSHR. By reversing the oxidative damage inflicted by H_2_O_2_, FME not only restored testosterone levels in TM3 Leydig cells but also enhanced their functional activity, surpassing even the effects observed with Testofen. Furthermore, the ability of FME to regulate enzymes like 5α-reductase and aromatase suggests it can regulate the balance of critical androgens and estrogens, addressing hormonal imbalances typical of LOH. The increase in AR and FSHR levels in Sertoli cells indicated the potential of FME to improve male reproductive health by enhancing spermatogenesis. Collectively, these findings indicate that FME is a promising therapeutic agent for combating LOH-related symptoms and improving overall male reproductive health. While the current study provides valuable insights into the effects of FME in vitro, further in vivo studies are required to fully understand its physiological relevance and potential therapeutic applications. Furthermore, the interaction between LH and LHR is a critical signaling pathway that initiates steroidogenesis in Leydig cells. In addition to changes in cell viability caused by H_2_O_2_-induced damage, further research focusing on LHR signaling appears to be equally important. Future studies should focus on clinical trials to validate these in vitro findings, as they are limited in replicating the complexity of physiological conditions. Furthermore, research should investigate the long-term benefits and safety of FME in vivo, addressing critical factors such as dose standardization and individual variability in treatment responses. With the growing interest in natural and safe alternatives to traditional hormone replacement therapies, FME emerges as a promising resource for mitigating the decline in male reproductive health.

## Figures and Tables

**Table 1:** Primer sequences for qRT-PCR.

Target Gene	GenBank Accession No.	Primer Sequences	Product Size (bp)
LHR	NM_013582.3	F: GATGCACAGTGGCACCTTC R: TCAGCGTGGCAACCAGTAG	185
StAR	NM_011485.5	F: GGAAGTCCCTCCAAGACTAAAC R: AGTCCTAGTGTCTCCTGACTAC	280
CYP11A1	NM_019779.4	F: GGGGCAACAAGCTGCCCTTCAA R: TGCAGGGTCATGGAGGTCGTGT	88
3β-HSD2	NM_153193.3	F: AGCAAAAAGATGGCTGAGAA R: GCACAAGTTTGCAAAGTGCC	80
17,20 desmolase	NM007809.3	F: AGAGTTTGCCATCCCGAAG R: AACTGGGTGTGGGTGTAATG	149
17β-HSD3	NM_008291.3	F: AATATGTCACGATCGGAGCTG R: GAAGGGATCCGGTTCAGAAT	78
5α-reductase	NM_053188.3	F: GCAAGCCTATTACCTGGTT R: AGAAGACACCGACGCTAA	79
aromatase	NM_007810.4	F: TGGACGAAAGTGCTATTGTGAA R: TCTTTCAAGTCCTTGACGGAT	136
AR	NM_013476.4	F: TTATGAAGCAGGGATGACTCTG R: GCTGCCAGCATTGGAGTT	93
FSHR	NM_013523.3	F: AGCAAGTTTGGCTGTTATGAGG R: GTTCTGGACTGAATGATTTAGAGG	155
GAPDH	NM_001001303.1	F: TCAACGGCACAGTCAAGG R: ACTCCACGACATACTCAGC	125

## Data Availability

Data are provided within the article.
